# More Than Just Gene Therapy Vectors: Lentiviral Vector Pseudotypes for Serological Investigation

**DOI:** 10.3390/v13020217

**Published:** 2021-01-31

**Authors:** Kamilla Toon, Emma M. Bentley, Giada Mattiuzzo

**Affiliations:** 1 Division of Virology, National Institute for Biological Standards and Control-MHRA, Blanche Lane, South Mimms EN6 3QG, UK; Kamilla.Toon@nibsc.org; 2 Division of Infection and Immunity, University College London, London WC1E 6BT, UK

**Keywords:** pseudotype, lentivirus, serology, vector

## Abstract

Serological assays detecting neutralising antibodies are important for determining the immune responses following infection or vaccination and are also often considered a correlate of protection. The target of neutralising antibodies is usually located in the Envelope protein on the viral surface, which mediates cell entry. As such, presentation of the Envelope protein on a lentiviral particle represents a convenient alternative to handling of a potentially high containment virus or for those viruses with no established cell culture system. The flexibility, relative safety and, in most cases, ease of production of lentiviral pseudotypes, have led to their use in serological assays for many applications such as the evaluation of candidate vaccines, screening and characterization of anti-viral therapeutics, and sero-surveillance. Above all, the speed of production of the lentiviral pseudotypes, once the envelope sequence is published, makes them important tools in the response to viral outbreaks, as shown during the COVID-19 pandemic in 2020. In this review, we provide an overview of the landscape of the serological applications of pseudotyped lentiviral vectors, with a brief discussion on their production and batch quality analysis. Finally, we evaluate their role as surrogates for the real virus and possible alternatives.

## 1. At a Glance: History of the Evolution of the Lentiviral Pseudotype System

**Figure 1:**
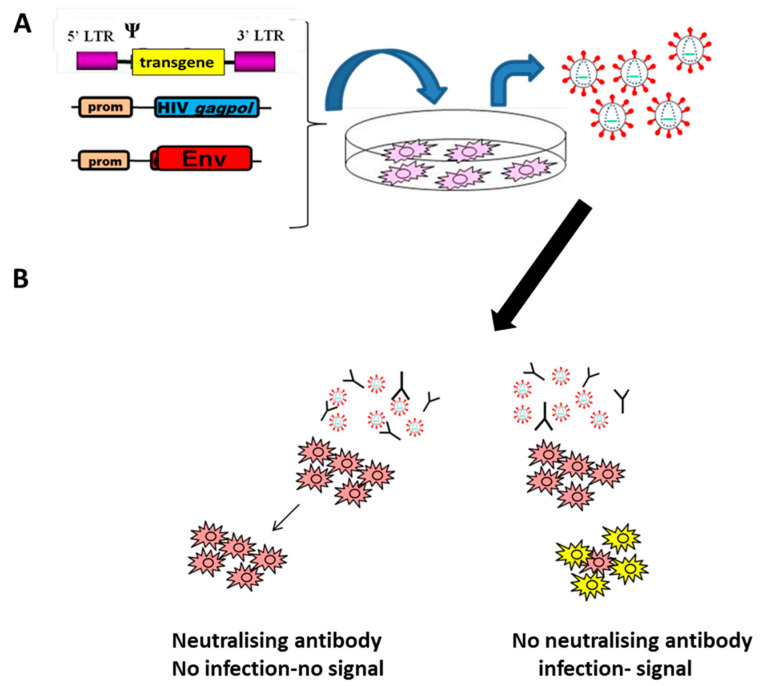
Application of pseudotyped lentiviral vectors (LVs) to neutralisation assays. Pseudotyped lentiviral particles are generated by transfection of a permissive cell line (e.g., HEK293T) with a mix of plasmids coding for HIV-1 structural and enzymatic proteins (HIV gagpol), the lentiviral vector with a transgene of interest, e.g., luciferase, GFP, contained within the HIV long terminal repeats (LTR) and an expression plasmid coding the Env of the virus of interest. Particles are collected in the supernatant of the transfected cells usually 48–72 h post-transfection (**A**). In a neutralisation assay, the pseudotyped LV particles are incubated with the serological sample to test before adding to the target cells. If no neutralising antibody is present, the pseudotyped LV will enter the target cells, and the transgene will be expressed, and infection can be monitored by the signal produced. Neutralising antibody in the sample will inhibit LV entry and no signal will be visible (**B**).

The first naturally observed pseudotype was a replication-defective Rous sarcoma virus (RSV) carrying on the particle surface the envelope protein (Env) of avian leukosis virus (ALV) [1]. The RSV genome had the envelope gene, *env*, replaced with an oncolytic gene, *src*. Co-infection of the same cells with ALV allowed the envelope-less RSV to acquire the ALV Env and infect cells, leading to malignancy. The pseudotyped viruses’ tropism at entry level and neutralisation properties are therefore determined by the Env on their surface, and not by the genetic information within the virus particles. Nowadays, this naturally occurring phenomenon has been engineered to serve many purposes such as gene therapy, virus–host interaction and serological studies. Many viruses have been exploited as a basis for a pseudotype [2,3], with lentiviral vectors (LVs) at the forefront [4,5,6]. LVs are mainly derived from human immunodeficiency virus type 1 (HIV-1), a single-stranded RNA virus from the *Retroviridae* family, genus *Lentivirus*. The relatively simple genome structure (less than 10,000 bases) is convenient for genetic manipulation [7]. Initially, the use of HIV-1 as a vector to deliver genes to a host cell, known as transgenes, was carried out with a replication-competent virus that had its own genes swapped for those of interest [8]. However, as HIV-1 is classified as a hazard group 3 pathogen, many modifications to the system have been made since to improve the safety of these vectors [9]. The most commonly used HIV-1 system has the viral genome split in three plasmids: the packaging plasmid containing the structural and essential accessory genes, the transfer plasmid containing the packaging signal (Ψ) for inclusion into the viral particles with the transgene to be integrated in the target cell and the third plasmid, which is for the expression of the Env which will determine the tropism of the viral particles (Figure 1a). A more detailed description of the molecular basis and evolution of the lentiviral vectors is provided in the review by Duvergé and Negroni in this Special Issue [5]. The other advantage of LVs is the lack of interaction between the core and Env for viral egress, with the capability to bud in the absence of Env [10,11] or more valuably incorporate a heterologous envelope [12]. The ability of an LV to have multiple different viral Envs “plugged in” to the surface makes it a flexible tool for gene delivery, basic viral research (e.g., viral entry, receptor studies), drug screening and serology (Figure 1b). This review focuses on the use of LVs in serological assays. LVs have proven to be a safe and efficient tool for screening for neutralising antibodies to a wide range of viral pathogens, especially as a substitute for pathogens designated hazard groups 3 and 4, where accessibility for research is restricted, especially in resource-limited areas where high-containment facilities are not available. Additionally, pseudotyped LVs play an important role in serological research for viruses that cannot be propagated in cell culture, such as most strains of hepatitis C virus or emerging viruses that do not have cell culture systems established yet [13,14].

## 2. Applications in Serological Studies

Serological assays are used for the evaluation of the immune response, specifically antibodies, in serum, plasma or other body fluids. Detection of pathogen-specific IgM can aid the diagnosis of acute infection in support of the preferred molecular methods detecting the genetic material (i.e., nucleic acid amplification-based techniques). This is particularly important for those infections characterised by a short viremia, such as Zika [15], or those in resource-limited areas. During the coronavirus disease 2019 (COVID-19) global pandemic in 2020, serology was considered a critical tool to understand the real impact of COVID-19, as many cases were asymptomatic or unreported, and to guide government policies [16,17]. Other applications of serological assays involving the use of pseudotyped LVs such as evaluation of vaccines and therapeutics are described in more detail below, and Table 1 is a summary of the LV serological applications discussed. There are many types of serological assays, which, in a simplistic manner, can be grouped into detection of either binding or neutralising antibodies. The enzyme-linked immunosorbent assay (ELISA) is the most traditional method for determining the presence of specific binding antibodies in a serological sample. Usually, recombinant proteins or peptides are the target antigens, but whole virus-based ELISAs have been developed [18,19,20], opening the possibility to replace the real virus with a pseudotyped LV, although restricting the ELISA specificity to the Env. The applications of pseudotyped LVs are more obvious in those assays that can detect neutralising antibodies (NAbs), as these are usually directed against the viral envelope proteins, which interact with the cellular receptor. Traditional assays such as the plaque reduction neutralisation test (PRNT) and hemagglutination inhibition (HI) assays require the use of a live virus, often at containment level 3 or 4 laboratories. Neutralisation assays which employ a pseudotyped LV offer the great advantage of a lower containment platform with a reduced risk for the scientist. The pseudotyped LV system allows laboratories which cannot afford the cost of a high-containment level laboratory, or which have difficulties in importing specific pathogens, to perform neutralisation assays. An added benefit of this system is that a marker gene such as green fluorescent protein (GFP) or luciferase (Luc), as a transgene, facilitates the infectivity readout, making the assay easier to perform and often more time-efficient. 

### 2.1. Evaluation of Vaccine Efficacy 

Detection and quantification of NAbs are considered the main correlates to evaluate the efficacy of vaccines, determine a protection titre and decipher the immune status of individuals to identify the risk of infection. For enveloped viruses, NAbs are primarily targeted towards the Env, blocking receptor binding and/or fusion and therefore the processes of entry and uncoating within the cell. They are often the best correlates of protection following vaccination [89]. During the ongoing development of a vaccine, assessment of its efficacy in producing a humoral immune response and the potency of NAbs against the Env in preventing infection is essential [90,91,92]. Pseudotyped LV-based neutralisation assays have been proven a useful tool to investigate NAbs raised by various vaccine candidates. During the evaluation of a viral vectored vaccine against Middle Eastern respiratory syndrome coronavirus (MERS-CoV), termed ChAdOx1 MERS, the NAb response was investigated by a pseudotyped LV-based neutralisation assay in preclinical studies in mice [23], through to a phase I clinical trial [24]. This included a parallel study determining the efficacy in a dromedary camel population [25]. The system was also applied to early vaccine studies for severe acute respiratory syndrome coronavirus (SARS-CoV) to identify NAbs raised to different Spike envelope protein fragments and to identify antigenic determinants to serve as potential vaccine targets [29,30]. Thus, it is of no surprise that pseudotyped LV-based neutralisation assays have played a key role in the COVID-19 global pandemic caused by the close relative SARS-CoV-2 [32,33,34]. The high-throughput platform assists timely evaluation of rapidly progressing clinical trials, employing upwards of 30,000 volunteers by phase III. 

There are many other examples in the emerging virus field where a pseudotyped LV has been used to investigate NAbs induced by candidate vaccines. Chikungunya virus-pseudotyped LVs have been used to determine the level of NAbs raised in non-human primates by a virus-like particle vaccine candidate and showed good correlation between the pseudotyped LV neutralisation method and the traditional PRNT with the live virus [88]; similar results were obtained using a Lassa fever virus pseudotyped LV for the evaluation of a candidate DNA vaccine in a non-human primate model [21]. This has further been demonstrated with Ebola virus pseudotyped LVs as part of a phase I clinical trial of a viral vectored vaccine regime [49]. Indeed, it is plausible that use of a pseudotyped LV to confirm the efficacy of the NAb response to vaccines under development will become commonplace in the emerging virus field. 

Following a different approach in the case of rabies virus vaccines, pseudotyped LVs have been exploited to evaluate levels of cross-neutralisation afforded by the vaccine to other antigenically diverse species within the Lyssavirus genus—through the screening of sera collected from vaccine recipients [80]. Constraints from the requirement for category 3 or 4 containment for handling lyssaviruses, together with accessibility to isolates from the 17 species identified within the genus, are overcome by the “plug and play” utility of the pseudotyping approach. This is also the case in the influenza field, with multiple studies reporting the humoral responses to numerous influenza A virus vaccine candidates [58,59,60,61,62,63,64,65,66]. A pseudotyped LV has proven particularly valuable in identifying neutralising antibodies to the less variable stalk region of the influenza HA by use of chimeric HA sequences which include a head region unreactive to antibodies under evaluation [93]. These have been applied to studies investigating novel stem-based protein immunogens, which offer a potential for development as a universal influenza vaccine [67,68,69]. In the case of both rabies and influenza virus, pseudotyped LV-based neutralisation assays have been shown as robust alternatives to current live virus gold standard assays for determining NAb levels, namely, the rapid fluorescent focus inhibition test (RFFIT) and microneutralisation (MN) assay, respectively [94]. Although the MN assay is commonly used in the evaluation of influenza virus vaccines, it has demonstrated significant inter-laboratory variability [95], in addition to the risk of working with highly pathogenic strains; as such, studies have shown pseudotyped LV neutralisation assays as a reliable alternative [96]. Further, a pseudotyped LV has been used as an alternative source of antigen for enzyme-linked lectin assays (ELLA), measuring antibodies raised to neuraminidase and alleviating complications of working with reassortant or detergent-treated viruses [97,98]. 

Vaccine development against hepatitis C virus (HCV) is complicated by a high level of genetic diversity, giving rise to multiple mechanisms of immune evasion [99], coupled with the lack of a preclinical animal model and cell culture systems for virus propagation [100]. The use of HCV pseudotyped LVs goes some way to addressing these shortfalls and was recently applied to measure the NAb response following a recombinant protein vaccination strategy during a phase I clinical trial against diverse strains of HCV, compared to that raised in a non-human primate and murine model [55]. This study identified non-human primates as an appropriate preclinical model and guides future development of vaccines towards epitopes affording broad neutralisation.

### 2.2. Serosurveillance Studies

Epidemiology studies provide the ability to understand a pathogen’s transmissibility (i.e., ability to spread in the population) virulence (i.e., severity of disease) impact on the community and to inform guidance for government policies and implementation of countermeasures. Epidemiology studies have focused primarily on the detection of antibodies as they remain in circulation long after the symptomatic stage of infection has occurred. Large serosurveys for emerging viruses have, for example, highlighted the longstanding disease burden of viruses circulating in Sierra Leone and the potentially high prevalence of Ebola virus antibodies in the population [50,51]. The inclusion of Ebola and Marburg pseudotyped LVs in such studies allowed a more specific analysis to find a lower predictive prevalence of filovirus antibodies than initially thought in individuals across five African countries [52,101]. Pseudotyped LVs have not only proven useful in large cross-population studies, but also within individuals; for HCV, they have been used to determine the time of seroconversion and investigate the role NAbs play at multiple timepoints through the course of infection [56]. Over the duration of 2020, there have been numerous publications using SARS-CoV-2-pseudotyped LV neutralisation assays in attempts to understand both the individual response and population exposure to the causative agent of COVID-19 [35,36,37,38,39]. It will be interesting to see whether this expansion of interest in the technology results in a subsequent increase in the application of pseudotyped LVs for the serosurveillance of other virus families. Another advantage presented by the LV system, which was highlighted during the COVID-19 pandemic, is the ability to rapidly respond to the emergence of new variants of concern; as soon as sequences are available, the new mutant spike protein can be incorporated onto the LV to evaluate the potential to escape neutralisation by convalescent and vaccinee sera or therapeutics [40,41,42,43] such as therapeutic monoclonal antibodies (mAbs) [44,45] from mutations arising in the spike protein.

With many enveloped viruses being zoonotic, a One Health approach is often applied to seroepidemiology studies. The detection of a high prevalence of MERS-CoV NAbs in dromedary camels compared to humans and other domestic livestock helped identify them as the potential reservoir in the Middle East region [26,27,28]. Influenza virus requires constant surveillance at the human–animal interface to identify the emergence of new subtypes and help guide seasonal vaccination campaigns, as well as monitoring population immunity. To this effect, pseudotyped LVs have been used in multiple studies [70,71,72,73,74]. The flexibility to incorporate different reporter genes within the LV was exploited in one study to produce a multiplex assay, simultaneously detecting NAbs against a H5 and H7 subtype in serum collected from vaccinated chickens [102]. The study also highlighted the applicability of pseudotyped LVs for in-field serology studies in endemic regions, with a requirement for only basic cold-chain storage. This has been supported by in-field lyssavirus serosurveillance studies in Africa of dog and bat populations by both standard and multiplex pseudotyped LV neutralisation assays [81,82]. Further advantages of the system highlighted by these studies include the requirement for only small serum volumes, typically 5–10-fold lower than in many traditional live virus neutralisation assays, as well as their relatively low cost and basic laboratory equipment requirements.

### 2.3. Cross-Reactive Antibody Responses

The outbreak of an arbovirus, Zika virus, in 2015 was recognised by the World Health Organization (WHO) as a Public Health Emergency of International Concern [103] and has raised awareness of the need to monitor these viruses. Neutralisation assays are required to distinguish between different arbovirus infections, as they induce cross-reactive antibodies within their family, such as *Flaviviridae* and *Togaviridae* [104,105]. Alphaviruses of the *Togaviridae* family, such as chikungunya virus (CHKV), Semliki Forest virus, Ross River virus and Mayaro virus, cause similar clinical symptoms, usually fever and long-lasting arthralgia. The pseudotyped LV platform not only offers the advantage of a lower containment level (e.g., CHKV is classified as hazard group 3) [88,106,107], but has also been expanded to include multiple alphavirus PVs to distinguish between alphaviruses’ reactivities [88]. This type of multiplex assay can also be used to isolate cross-reactive neutralising antibodies and to produce broad-spectrum monoclonal antibodies as therapeutics, or to identify critical conserved epitopes to guide vaccine design. A similar approach was proposed by Luczkowiak and colleagues, by using a filovirus pseudotyped LV to investigate the cross-neutralising activity of convalescent sera from Ebola virus disease recovered patients against other members of the genus *Ebolavirus*. They identified cross-reactive potent NAb epitopes hidden by a glycan shield in the mucin-like domain of the glycoprotein, leading to the suggestion that removal of these regions in a candidate vaccine could increase the induction of NAbs [53]. Viral escape from NAbs by glycan shielding in the haemagglutinin, with the same implication for vaccine design, was also proposed for influenza virus; in this study, pseudotyped LV-based assays assessed the neutralising antibody titres in support of the gold standard HI method [108].

### 2.4. Antigenic Site Analysis

A further advantage of the inherent flexibility provided by the pseudotype system has been in the ability to easily manipulate the Env incorporated on the LV, which can be exploited to identify NAb epitopes. For instance, epitope specificity of candidate therapeutic monoclonal antibodies (mAbs) for the post-exposure prophylaxis of rabies virus has been characterised by displaying an Env with mutated antigenic sites on the pseudotyped LV and evaluating the effect on neutralisation [83,84]. Taking this further, a study by Evans et al. [85] switched each of the five defined antigenic sites of the lyssavirus Env between divergent species to investigate the effect on neutralisation by polyclonal sera; this allowed the identification of those important for broad neutralisation, with implications for vaccine design. A similar study but with a different objective was conducted by displaying mutant and chimeric vesicular stomatitis virus (VSV) glycoprotein variants on a pseudotyped LV to define the binding epitope and mechanism of neutralisation of commercially available mAbs; this mAb can now be employed as a tool for the characterisation of those pseudotypes using the vesiculovirus Env in academic and clinical settings [86]. Other studies, in contrast, have looked to identify escape mutants: pre-existing immunity to measles virus (MV) glycoproteins induced by vaccination presents a drawback to the use of these envelope proteins in gene therapy, such as to target lymphocyte cells. Pseudotyped LVs incorporating the MV glycoprotein with target mutations and augmentations have been found to be resistant to neutralisation by MV-specific antibody-positive human serum [75,76], which may offer a route towards their implementation in gene therapy and immunotherapy. Of note, an LV pseudotyped with the Nipah virus glycoprotein has been suggested as a better option to the MV-LV as it is not affected by widespread acquired or vaccine-induced immunity to MV and can be produced at a higher titre [109].

### 2.5. Selection of Therapeutic Antivirals

One of the attractive properties of pseudotypes comes from their amenability to a high-throughput setting, which is particularly useful for the discovery of drugs inhibiting viral entry. In the case of emerging viruses, this has involved screening existing licensed medicines for the potential to be repurposed in the event of an outbreak. A study by Wang et al. [110] described the design of experiments to screen against multiple pseudotyped LVs simultaneously. Indeed, such an approach was applied in two studies, with one screening more than 1000 approved medicines, confirming chloroquine as a promising candidate against both Ebola and Marburg viruses, while also evaluating candidates against avian influenza or Lassa virus [22,54]. One study took a different approach, reporting the use of a dual Env pseudotyped LV to identify inhibitors of both SARS-CoV entry and particle assembly, with the second Env reducing false positives [31]. Multiple studies have looked to identify potent and broadly neutralising monoclonal antibodies (mAb) with therapeutic potential. For instance, panels of HIV-1-pseudotyped LVs displaying 118 Env isolates across all known clades were used in the identification of mAb 3BNC117 which has since progressed through phase II clinical trials [77,78,79]. For HCV, a panel of 78 patient-derived envelope protein E1E2 clones were used to pseudotype LVs and evaluate antibody efficacy against circulating HCV strains; the study showed a high degree of variability in the neutralisation sensitivity of the patient-derived E1E2, highlighting how the use of a panel of (pseudotyped) viruses will be an essential tool to truly investigate the efficacy of therapeutic mAbs [57]. Most recently, in response to the COVID-19 pandemic, SARS-CoV-2-pseudotyped LV-based neutralisation assays were used to determine the potency of a mAb cocktail as a candidate therapeutic strategy which was rapidly progressed into a phase I clinical trial [46,47]. Another therapeutic approach which has been evaluated for COVID-19 is the use of convalescent plasma from recovered patients. The pseudotyped LV system has provided a high-throughput platform for the evaluation of the neutralising antibody titre in the plasma donations [48]. These studies act to exemplify the flexibilities the pseudotyping system has to offer.

## 3. Production of Pseudotyped LVs

### 3.1. Factors towards Optimising Titres

Despite many protocols having been published describing the production and titration of pseudotyped LVs [111,112,113,114,115,116], many variations exist with seemingly subtle changes having a big impact on production efficiency. Even within a viral family, optimal protocols or production efficiencies may differ. Although protocols had been published on producing MERS-CoV [117] and SARS-CoV pseudotyped [118] LVs used within our laboratories, when SARS-CoV-2 emerged, it still took many weeks to establish a reliable approach to produce workable titres. Ultimately, the target cell line proved to be an important factor, with over-expression of the human receptor ACE-2 and, in some cases, the cellular protease TMPRSS2 increasing titres to a workable level [119,120,121]. It was also found that truncation of the spike envelope protein could increase titres further. This has previously been demonstrated for pseudotyped LVs bearing the fusion protein of Nipah and Hendra paramyxoviruses [122], as well as measles virus [123]. It has also been shown that comparatively low titres of a rabies pseudotyped LV could be increased using a chimeric envelope protein with the cytoplasmic domain switched for that of the VSV glycoprotein, which is known for generating high-titre pseudotyped LVs [124]. This was similarly found for the feline endogenous retrovirus RD114, with titres improved by altering the cytoplasmic domain to an alternate retrovirus [125]. The mechanism behind these increases is not fully understood and speculated to be due to more efficient incorporation onto the LV core. The density of the Env on the LV core in comparison to the wild-type virus remains a resounding question amongst the pseudotyping community. Visual inspection of particles by electron microscopy may offer one route to a better understanding, yet it is insensitive and technically challenging which might explain the lack of data reported on this issue. New technologies based on fluorescent microscopy, such as stimulated emission depletion (STED) microscopy [126], could provide a more approachable method to investigate the density of Env molecules on LV particles.

Other factors to consider when optimising pseudotyped LV production include the choice of the envelope expression plasmid and the ratio used alongside those containing the packaging and reporter elements, as well as the transfection reagent for delivery to the producer cell. The importance of applying an empirical approach to optimising total plasmid amount and ratios for each specific Env of study has been well demonstrated by Urbanowicz et al. [14], for hepatitis C virus and Ebola virus. A previous study also showed that lower levels of input of the Ebola virus envelope protein increased titres, suggesting higher levels may impair processing and expression by cellular machinery and that LVs densely packed with the envelope could result in impaired receptor binding [127]. Other technical aspects of production have been discussed as part of a systematic review of influenza pseudotyped LV production [93], which is further complicated by the requirement to provide an exogenous protease to proteolytically cleave the haemagglutinin envelope protein during pseudotyped LV production for it to become fusion-competent [128].

### 3.2. Quantification of LV Yield

Reporter genes are incorporated within the LV as an RNA dimer which is reverse transcribed and expressed following integration into the target cell genome post-entry. Multiple reporter genes have been incorporated within the pseudotype platform [129], yet β-galactosidase (β-gal), fluorescent proteins (e.g., green fluorescent protein, GFP) and luciferase are most used with good levels of sensitivity and high-throughput acquisition. Infectious titres of the pseudotyped LV stock must be determined for normalisation of the downstream assay input. Infectivity of LVs containing the genes for β-gal or GFP is detected by colorimetric stain (β-gal) or fluorescence (GFP) visualised on a fluorescent microscope or acquired by flow cytometry. In both cases, the titre is expressed in infectious units (IFU), calculating the number of infected cells per volume of infection dose. Luciferase activity is instead quantified as relative light units (RLU) by the detection of luminescence after complete lysis of the cells in a well; the numerical value of the RLU may vary considerably between laboratories, mainly due to the use of different luminometers. To circumvent this issue, titres should be determined via endpoint serial dilutions, following standard virological practice, to report the 50% tissue culture infectious dose (TCID_50_) [130,131]. This allows comparability and reproducibility across studies as well as between the disparate readout units from alternative reporter genes.

Detection of the reporter gene signal from pseudotyped LV-infected cells provides a functional titre; however, other non-functional measurements have been used to quantify the LV stock and normalise the viral input in downstream applications. The most commonly used method is determining the amount of the LV core protein by antigen capture ELISA measuring HIV p24 levels. It is accepted in the field that 1 ng of p24 will correspond to 1000–5000 particles [132,133]. Several commercial kits are available, and it is the least time-consuming method. LV particles can also be quantified by measuring the reverse transcriptase (RT) activity associated with the core component: several commercial kits and in-house methods are available, such as an SYBR Green product-enhanced RT (SG-PERT) assay [134,135]. Quantification of the LV titre can further be achieved by measuring the amount of viral genomes; as the viral genome is RNA, this can be performed by quantitative reverse transcriptase polymerase chain reaction (qRT-PCR) [136]. More recently, a novel competitive qRT-PCR has been developed to improve the quantification of the viral genomic RNA packaged within the LV [137]. Finally, total particle content can be determined by nanoparticle tracking system analysis [138,139] and flow-based instruments [140]. These methods require specialised equipment but provide important information on the quality of the preparation by providing a ratio of infectious to total particles. Although these measures will be greater than the functional titres [139,141,142,143,144], they should not be overlooked as an important adjunct to functional titre evaluation towards standardising the quality of pseudotyped LV production.

### 3.3. Quality Control of LVs 

Quantification of the infectious viral particles, i.e., transducing particles, and ratio with the total amount of particles in the stock preparation is one of the factors which should be considered when looking at the quality of a batch of LVs, as listed in the guidelines on the development and manufacture of LV published by the European Medicine Agency [145]. This document targets the large-scale manufacturing of LVs for clinical trials, usually for gene therapy; however, it can be useful to help define the critical parameters for the quality control of pseudotyped LVs for in vitro applications. For instance, an important safety issue mentioned is the testing for recombination-competent lentiviruses (RCL); while this is obviously critical for a medical product for human use, it is also a health and safety requirement for the production of LVs for research or preclinical applications and should be included in all risk assessments covering the production and use of pseudotyped LVs. Methods for testing RCL for clinical-grade products are available [146], and so far, using the latest generation of lentiviral systems, no RCL has been reported even in large-scale production [147,148]. The need for high-quality LVs for clinical applications has led to the publication of standard operating procedures for production of LVs [149,150,151,152] and many companies offer Good Manufacturing Practice (GMP)-grade LV preparations (e.g., Patheon by Thermo Fisher Scientific, Lonza Pharma and BioTech, ProMab Biotechnologies, Inc., Flash therapeutics, etc.). For most of the serological applications of pseudotyped LVs, this level of quality assurance may not be required. However, should the data produced by pseudotyped LV-based neutralisation assays be used for licensing of a medical product (e.g., to prove the efficacy of a therapeutics such as mAbs, immune response elicited by a candidate vaccine), it is recommended that the laboratories performing the test should work following good laboratory practice [153], with traceability of the production of the LVs and batch to batch consistency. The use of a common reference material can greatly improve the comparability of assays and allows for monitoring assay performance in time; the WHO International Standards are the highest order of reference reagents and the primary calibrators of assays. Their use for pre-marketing applications is recommended [154]. For LVs, an example of such material is the WHO International Reference Reagent for the quantification of lentiviral vector integration [155,156]. A similar reference material could be used in serology assays as quality control for each pseudotyped LV batch; however, while a generic standard could be made to standardise the physical quantification of the particles, a specific reference material is needed for every Env used for the pseudotype. Further, these reference preparations should be suitably stable for a long period of time; for instance, WHO guidelines for an international standard suggest a period of 5–10 years [157]. To ensure the stability of the LVs, lyophilisation has been described as an optimal solution [158,159].

## 4. Correlation with Live Virus Assays

Optimisation of the production of pseudotyped LVs should also take into account the downstream application of these particles. For serological assays, it is important that the antibody response in a sample is the same, or at least correlates, between assays employing pseudotyped LVs and those using the real virus. There are many examples in the literature showing a good correlation in neutralisation assays. Wright et al. demonstrated that rabies antibody titres in over 190 Tanzanian dogs obtained from a pseudotyped LV- based neutralisation assay correlate strongly with the fluorescent antibody virus neutralisation (FAVN) live virus assay, r = 0.915 [81]. Similarly, good correlation coefficients were obtained in studies evaluating NAbs in dromedary camel populations in Saudi Arabia for MERS, r = 0.88, and in 23 CHIKV patients in Thailand, r^2^ = 0.78 [27,107]. Good concordance was also observed in the NAb titres between a SARS-CoV-2-pseudotyped LV and a live virus microneutralisation assay (MN) during the evaluation of 65 samples from an Italian sero-epidemiological study [160] and of convalescent plasma from 52 donors in the United Kingdom [48]. Alberini and colleagues analysed the correlation of titres from a phase 2 clinical trial for a candidate flu vaccine between influenza H5N1-pseudotyped LV neutralisation assays and three traditional serological assays for detection of anti-HA antibodies, haemagglutinin inhibition (HI), single radial haemolysis (SRH) and MN assays, resulting in good correlations of r = 0.73, 0.70 and 0.78, respectively [96]. However, replacement of a live virus neutralisation assay with a pseudotyped LV is not always straightforward. For instance, the correlation between the HI assay and influenza A pseudotyped LV neutralisation assay has been shown to vary from poor (r = 0.1171, A/Japan/305/57(H2N2)) to strong (r = 0.8454, A/Brisbane/59/2007(H1N1)) depending on the HA subtype displayed on the PV [161]. Moreover, for Ebola virus (EBOV), two independent studies showed that pseudotyped neutralisation assays where the EBOV glycoprotein is expressed on a recombinant vesicular stomatitis virus (VSV) vector correlate better to the live neutralisation assay than an EBOV pseudotyped LV [162,163]. A caveat of both studies is that the pseudotyped VSV and live virus neutralisation assays used the same target cells (African green monkey fibroblast cell line, VERO), whilst the pseudotyped LV assays were conducted on a human embryonic epithelia cell line, HEK293T.

## 5. Alternative Vector Systems

There are several examples of viruses whose genome has been manipulated to create pseudotyped vectors. As mentioned in the previous paragraph, the VSV pseudotyping platform has become increasingly popular [2]. A benefit of this platform over the LV is the cytoplasmic VSV replication [164], which reduces assay times as reporter expression can be detected within 24 h after transduction. Additionally, the appropriate target cell lines are crucial to establishing a functional pseudotype neutralisation assay; thus, vector backbone selection hinges on whether the required cell lines allow infection by the vector. For example, all monkey-derived cell lines (e.g., VERO, COS) are resistant to HIV-1 infection due to expression of restriction factors, such as TRIM5α [165]. While LV-based fusion assays can overcome post-entry issues [166], so does the use of other viral vectors such as VSV. Another alternative is the use of a different retrovirus, murine leukaemia virus (MLV); this has been widely used as a viral vector system in both gene therapy and for serological assays. Despite sharing many similarities with the LVs, the Env from some viruses will pseudotype onto one system and not another, which is demonstrated by multiple HCV isolate Envs that failed to pseudotype an LV but became functional on the MLV vectors [14]. The reasons for preferential pseudotyping onto one retroviral backbone and not another are not completely understood but are likely linked to subtle differences in viral assembly sites which effect colocalisation with the Env [167,168]. Another virus engineered to serve as a pseudotyped vector for serological assays is influenza virus; this system has been used to express the Ebola virus glycoprotein for the selection of therapeutic mAbs [169]. These are only some examples of viruses which have been manipulated to create pseudotyped vectors, but many others have been used as the basis of a pseudotype for research use. 

The Envs of some viruses have been difficult to nearly impossible to pseudotype into the above-mentioned platforms—for instance, many members of the *Flavivirus* genus such as West Nile virus (WNV) and Japanese encephalitis virus [94]. The issue lies in the virus life cycle; these are internally budding viruses that acquire their envelope lipidic membrane from the cytoplasmic membrane system (endoplasmic reticulum, Golgi apparatus), whilst the main site of budding for retroviruses and VSV is the plasma membrane. As such, the Envs of internally budding viruses are not in the required location (cell membrane), where the retroviral or VSV particles bud. Research into other platforms has been undertaken to establish alternative backbones for internally budding viruses. Pierson et al. produced a sub-genomic replicon with the non-structural proteins of WNV incorporating a GFP or luciferase reporter gene. When complemented in trans with plasmids expressing WNV structural proteins, single-round replication reporter virus particles (RVP) were generated. These RVP were able to incorporate multiple strains of WNV [170]. Similar approaches have been developed using dengue virus reporter replicons to produce particles with multiple heterologous flavivirus Envs on their surface [171,172]. The uptake of these new systems gives way to a whole new era of pseudotyping.

## 6. Conclusions

Over the course of the last two decades, pseudotyped LVs have cemented themselves as a robust alternative to handling live virus isolates for serological investigations. Studies have exploited unique flexibilities in their design and construction to advance knowledge in challenging areas, overcoming constraints on resources, the need for high-containment facilities or the lack of cell culture systems. While taking note of where we stand today, it is evident that there is an increasing uptake of pseudotyped LVs; as exemplified by the record number of studies throughout 2020 which have widely reported their use in response to the emergence of SARS-CoV-2. We foresee there will be a benefit and need for enhanced characterisation of pseudotyped LV stocks in support of serological investigations supporting medicinal product licensure. Further, enhanced methods of quantification will play a role in comparisons against other novel vector systems, assisting identification of the most appropriate platform for each Env. LVs will act as a prototype in this continued trend towards pseudotype-based serology.

## Figures and Tables

**Table 1:** Serological applications of pseudotyped lentiviral vectors.

Virus Family	Virus Envelope Protein	Serological Application
***Arenaviridae***	Lassa fever virus G-protein	Vaccine Efficacy [21] Therapeutic Antivirals [22]
***Coronaviridae***	Middle East respiratory syndrome coronavirus S-protein	Vaccine Efficacy [23,24,25] Serosurveillance [26,27,28]
	Severe acute respiratory syndrome coronavirus S-protein	Vaccine Efficacy [29,30] Therapeutic Antivirals [31]
	Sever acute respiratory syndrome coronavirus-2 S-protein	Vaccine Efficacy [32,33,34] Serosurveillance [35,36,37,38,39,40,41,42,43,44,45] Therapeutic Antivirals [46,47,48]
***Filoviridae***	Ebola virus G-protein	Vaccine Efficacy [49] Serosurveillance [50,51,52]
	Ebola, Sudan, Bundibugyo, Reston virus G-protein	Cross-Reactivity [53]
	Ebola, Marburg virus G-protein	Therapeutic Antivirals [22,54]
***Flaviviridae***	Hepatitis C virus E1/E2-protein	Vaccine Efficacy [55] Serosurveillance [56] Therapeutic Antivirals [57]
***Orthomyxoviridae***	Influenza A virus HA and NA-protein	Vaccine Efficacy [58,59,60,61,62,63,64,65,66,67,68,69] Serosurveillance [70,71,72,73,74] Therapeutic Antivirals [54]
***Paramyxoviridae***	Measles virus H-protein	Antigenic Site Analysis [75,76]
***Retroviridae***	Human immunodeficiency virus 1 G-protein	Therapeutic Antivirals [77,78,79]
***Rhabdoviridae***	Rabies, European bat 1, European bat 2 lyssavirus G-protein	Vaccine Efficacy [80]
	Rabies, Lagos bat, Duvenhage, Mokola, West Caucasian bat lyssavirus G-protein	Serosurveillance [81,82]
	Rabies lyssavirus, vesicular stomatitis virus G-protein	Antigenic Site Analysis [83,84,85,86]
***Togaviridae***	Chikungunya virus E1/E2-protein	Vaccine Efficacy [87]
	Chikungunya, Mayaro, Ross River, Semliki Forest, Barmah Forest, O’nyong nyong virus E1/E2-protein	Cross-Reactivity [88]

## Data Availability

No new data were created or analysed in this study. Data sharing is not applicable to this article.
